# *In vitro* and *in vivo* evaluation of antibacterial and anti-biofilm properties of five ethnomedicinal plants against oral bacteria by TEM 

**Published:** 2021

**Authors:** Fariba Fathi, Maryam Sadrnia, Mohammad Arjomandzadegan, Hamid Reza Mohajerani

**Affiliations:** 1 *Department of Biology, Arak Branch, Islamic Azad University, Arak, Iran*; 2 *Department of Biology, Payame Noor University, Iran*; 3 *Department of Microbiology, Arak Branch, Islamic Azad University, Arak, Iran*; 4 *Infectious Diseases Research Center, Arak University of Medical Sciences, Arak, Iran*; 5 *Department of Biology, Arak Branch, Islamic Azad University, Arak, Iran*

**Keywords:** Ethnomedicinalplants, Antibacterial, Anti-biofilm, Oral bacteria

## Abstract

**Objective:**

The aim of the present study was to investigate antibacterial and antibiofilm activity of a few medicinal plants against oral bacteria.

**Materials and Methods:**

*Salvia officinalis*,* Lippie citriodora, Mentha piperita*,* Echinacea purpurea* and *Matricaria chamomilla* were extracted. Isolates from oral cavity were identified by microbiological and molecular methods. Minimum inhibitory concentration and minimum bactericidal concentration were determined by Broth microdilution method. The anti-biofilm activity of essential oils and extracts investigated and as a mixture by Broth dilution method. Toxicity of the herbal mixture was assayed by in Wistar rats treated with intradermal injection. Wound healing properties of the herbal mixture against infected wounds on the back of the rats were investigated. Anti-biofilm activity was investigated on tooth surfaces. Bacterial structure changes and fine- structure study were performed by light microscopy and Transmission electron microscopy.

**Results:**

The lowest MIC and MBC for the plant mixtures was 0.0002 mg/ml belonged to *Streptococcus pyogenes* and the highest values (0.025 mg/ml) belonged to *Eikenella corrodens*. The essential oils of *S.officinalis*, *L.citriodora* and *M.piperita, *but not *E.purpurea* and *M.chamomilla* extracts, were able to remove the biofilms created by the studied bacteria. The herbal mixture was able to completely heal the wound skin of rats in 21 days (p<0.05 compared to control). The mixture was able to decompose the teeth biofilm in 45 seconds. The results of light and electron microscopy showed that the bacterial structure exposed to the herbal mixture was deformed.

**Conclusion:**

It was concluded that the essential oils of *S.officinalis, L.citriodora* and *M.piperita* had significant effects on inhibition of oral bacteria biofilm formation.

## Introduction

Tooth decay is one of the most common infectious diseases in humans. The most important cause of tooth decay is the attachment of oral bacteria especially *Streptococcus* to different oral surfaces. To do this, these bacteria first attach to the thin salivary membrane by adhesions proteins and accumulate in the biofilm in the presence of sucrose and glucans synthesized by Glucosyltransferases, eventually producing acid and creating a low-pH environment (Ferreira et al., 2020 ▶)(Chi, 2013 ▶). Given the importance of binding properties of these bacteria and their ability to form biofilm that ultimately provides the basis for the destruction of tooth enamel, it is essential to find natural substances capable of inhibiting biofilm formation.

Both Gram-positive and Gram-negative bacteria can produce biofilms. Among the most important bacteria capable of biofilm formation, we can note Gram-positive bacteria such as *Enterococcus faecalis*, *Staphylococcus aureus, Staphylococcus epidermidis *and *Streptococcus viridans* and Gram-negative bacteria such as *Escherichia coli*, *Klebsiella pneumoniae*, *Proteus mirabilis* and *Pseudomonas aeruginosa* (Ulloa-Urizar et al., 2015 ▶).

Biofilm formation is a very fast process that always involves several steps; also, active participation of microbial cells is essential for adhesion. Production of extracellular polymeric materials is of great importance for biofilm formation and surface structures such as pili and flagella (Chen and Wang, 2010 ▶). Biofilms from pathogenic microorganisms play an important role in human health and usually, they do not respond well to treatment due to their resistance to detergents and antimicrobial agents. Moreover, the unnecessary increase in the use of antibiotics in health-care centers or by people who self-prescribe medications, can lead to multidrug-resistant infectious diseases. Identification and evaluation of antimicrobial compounds in extracts and essential oils of medicinal plants as new drug against this challenge, seem to be important (Ghasemi et al., 2012 ▶). Maintaining oral hygiene and using a toothbrush and mouthwash prevent bacterial growth, plaque formation and tooth decay (Das and Tiwari, 2010 ▶). After years of using chemicals as mouthwashes such as chlorhexidine and triclosan, their replacement with natural extracts and herbal remedies has become a priority (Khorasani et al., 1367).

 A long history of using herbs in traditional medicine and identifying many of their therapeutic properties over the years, as well as general acceptance, easy access, low cost of production and no environmental problems have made them suitable options for the control of resistant microbial strains (Khokhini et al., 1371 ▶).

According to Iranian traditional medicine, finding new sources of this knowledge in the treatment of oral diseases seems necessary. *Salvia officinalis*,* Lippie citriodora, Mentha piperita*,*Echinacea purpurea* and *Matricaria chamomila* are among the most commonly used plants (Sokmen et al, 1999 ▶). These plants were studies in the present work to provide a low-risk solution especially for people with dry mouth or those undergoing treatments such as radiotherapy with extensive and uncontrolled caries; thus,in this study, anti-biofilm effects of these plants on tooth decay bacteria, were assessed.

*M. chamomilla* is a one-year-old aroma plant from the chicory family. It grows in farms and gardens in central and southern Iran (Vahidi, 1986 ▶). Shiraz M.chamomilla contains various substances such as kamazolen, flavonoids that cause anti-inflammatory, antispasmodic, antimicrobial, antifungal, palliative and wound healing effects and accelerate wound healing (Arazi, 2003 ▶)(Khalessi et al.,2004 ▶).

 Due to the importance of bacterial colonization on the tooth surface and the destructive role of attachment and adhesion of these bacteria to the tooth surfaces and considering the antibiotic resistance, it seems beneficial to apply natural materials that have the potential to reduce the bonding of tooth decay bacteria.

*Salvia officinalis* as a herbaceous and perennial plant, is one of the well-known herbs in the herbal and food industry. Its application in anorexia, inflammation of the mouth and pharynx, and increased perspiration was approved by herbal medicine authorities. The decoction of the leaves is used as a gargle to cleanse and disinfect the mouth and to heal mouth sores and pharyngeal disorders.

*Mentha piperita* is a herbaceous and perennial plant. Peppermint is one of the medicinal plants that has attracted the attention of researchers due to its numerous medicinal effects (Fazel, 2004 ▶). According to a previous study, menthol is the most commonly used compound of mint, which is an important disinfectant and has marked antibiotic effects; also, it can cause a cool sensation in the skin and mucosa (Chevallier, 2003 ▶).

*Echinacea purpurea* is widely used in pharmaceutical, food, cosmetics and health industries. Although it is not native to Iran, it has been introduced to native Iranian plants in recent years. It has antifungal, anti-bacterial, and anti-viral properties and is used to prevent and treat colds, bronchitis, lung infections, cutaneous disorders and chronic immune response deficiencies (Bauer et al., 1988 ▶). In recent years, the antimicrobial properties of this plant were recognized and attributed to the presence of phenolic compounds (Hu and Kitts, 2000 ▶).

Blossom, scientifically named *Lippia citriodora*, is a shrub from the herbaceous family Verbenaceae, that reaches 3 to 5 meters in height. The leaves and vegetative organs of this plant have anti-fever, analgesic, antifungal, digestive and sedative properties and are used in the treatment of colds and headaches.

Given the relative resistance to biocidal agents as well as antibiotics among natural oral microflora especially *Streptococcus viridans* and recent research on the use of herbal medicine, this study was designed to evaluate the antibacterial activity of essential oil of a few herbal materials on oral bacteria.

## Materials and Methods

**Isolation of microorganisms**

Samples from oral cavity, tongue tip, and gingival groove were collected using a sterile swab. The specimens were cultured on a Mueller-Hinton agar medium (Merck, KGaA) containing 5% sheep blood and incubated at 37°C for 18 to 24 hr. Bacteria were isolated and diagnosed on the basis of colony morphology, Gram staining, and biochemical tests. Furthermore, four standard bacterial strains *Actinomyces viscousus* (PTCC1202), *Eikenella corrodens* (PTCC1391), *Streptococcus sanguis* (PTCC1449), and *Streptococcus viridans* (PTCC1774) from Iranian Scientific and Industrial Research Organization, were prepared and incubated in Brain Heart Infusion Broth at 37°C. 

**Essential oil preparation**

Essential oils of *S. officinalis, M. piperita *and *C. lippia* were prepared as follows: 30 g of the plant powder was put into a Clevenger apparatus and 300 ml of distilled water was added. The suspension was heated up to 80°C the mixed for 2 hr and the essence was stored in a dark glass container in a refrigerator at 4°C. 

**Extract preparation**

The aqueous extract of *E. purpurea* and *S. officinalis* was prepared by pouring 400 g of crushed herbs in 500 ml of water and heating the suspension for 6 hr. The extract was then stored in a dark glass container in a refrigerator at 4°C. 

**Acute toxicity**

Forty-two rats were randomly divided into six equal groups (7 in each). The control group did not receive any extract or essential oil.

The groups received 100, 500, 1000, 1250 and 1500 times the essential oils or extracts were injected intraperitoneally (Cockerill, 2006). One week after injection of the extracts or essential oils, mortality of the studied animals was assayed.

**Molecular identification of the isolates**

Two primer pairs for the *rpoA* gene were designed for *S. pyogenes* and the *rpoB* gene for *E. faecalis *(Table 1). Melting temperature, loop and other properties of the primers were examined by molecular software (DNASIS, BLAS, Oligo).

**Table1:** Primer specifications

Sequence	Primer
GACAAAACCCAGTCGTTGCT	*F-rpoA S.pyogenes*
TGAGGGCTTCTTCACCAACA	*R-rpoA S.pyogenes*
TCGATATTACAGGACCCGCT	*F.rpoB E.faecalis*
ACATAGCCACGACCAGGTTT	*R.rpoB E.faecalis*

DNA polymerase Taq, restriction enzymes, DNA ladder (100 base pair), ethidium bromide, etc. were prepared from Fanavaran gene company (Iran). The reaction was carried out at a final volume of 25 µl. In this reaction, 1 µlof the template DNA, 5 µlof DNA polymerase taq (5 units per microliter), 0.5 ml of each initiator, 2 µlof the mixed dNTPs (2.5 mM), 2.5 µlPCR buffer (10 x) and 1.5 µlof MgCl_2_ salt (50 mM) were mixed together; the final volume was set at 25 µl using distilled water. The PCR program was performed at 35 cycles (Table 2). Finally, 5 µl of reaction products was examined for any bonds in 2% agarose gel for 15 min under the 100 kev by electrophoresis.

**Wound healing study**

Wound healing properties of the herbal mixture were assessed by the following method. Anesthesia using ketamine and xylazine was induced. Back hair was shaved and disinfected; then, using circular marker and ruler, circles of approximately 2 cm in diameter, were drawn on the back of the animal. The skin was completely removed by a razor blade, so that the depth of the wound included dermis and epidermis. Wounds were taken care of and the animals were kept in separate cages. Animals were randomly divided into 3 groups of 6. The first group was the control group that received no treatment, the second group was daily treated with Vaseline ointment twice daily, and the third group was daily treated with the herbal mixture 2 times daily at specific times (every 12hr) for a period of 7, 14, 21 and 30 days.

**Table2:** PCR program

Temperature	Time	Steps
95˚C	5 min	Initial Denaturation
95˚C	30 sec	Denaturation
59˚C	50 sec	Annealing
72˚C	80 sec	Extension
72˚C	7 min	Final Extension

**Anti-biofilm assays**

Coffey method was accomplished as follows: A sterile 96-well microtitre polystyrene plate was used to evaluate the inhibitory effect of 10 microliter herbal materials (in 0.1 mg/ml concentration) on biofilm-producing bacteria. First, the bacterium was prepared equal 0.5 McFarland standards, then, BHI broth medium (Germany, Merck) with 2% sucrose was added. Next, the bacteria were inoculated into the wells and then the test substances were added to each well at different concentrations and placed in 37°C incubator for 48 hr. Herbal material and bacteria were considered positive and negative controls in the 11^th^ and 12^th^ wells respectively. After 48hr. of incubation, all wells were slowly emptied, and to stabilize the bacterial biofilm, 200 µl of pure methanol was added to the wells for 10 min and kept at room temperature. After emptying, the crystal violet was added and exposed to this color and room temperature for 20 min and rinsed with DW. Finally, Absorbance was read at 550 nm using a spectrophotometer (Coffey and Anderson, 2014 ▶).

**Determination of minimum inhibitory concentration**

Determination of MIC was carried out in 96-well plates. For this purpose, 100 µlof bacterial suspension equivalent to 0.5 McFarland was added to all wells of the plates. Then, 100 µl of the plant mixture was added the first well and completely sampled and then, 100 µl from the first well was removed and added to the next well. This procedure went on until well #10. Well No. 11 (positive control) contained 100 µl of bacterial culture medium. Since the extracts are generally colored and cause an optical absorption reading error, well No. 12 (control) contained 100 µlof pure extract or essential oil for determination of the optical absorption of the extract or essence. Optical absorption for all microplates were read at the 545 nm wavelength using an ELISA reader (Coffey and Anderson, 2014 ▶).

.

**Light microscopy**

The mixture of plants materials was assayed against 7 oral microbes for 0, 1, 2, 3 and 4 hours. Gram stained samples were observed by a light microscope (at 100X) to determine the time of bacterial destruction.

**Electron microscopy**

Based on the light microscopy results, one of the bacteria was randomly selected and when the changes were observed by light microscopy, the same exposure time was considered for examination by electron microscopy. For this purpose, the test was repeated at the determined concentration of the bacterium and the herbal material. The sample was centrifuged for 4 min at 2000 rpm in 1hr exposure time. The precipitate from the centrifuge was separated from its supernatant and washed using physiological serum and distilled water at 50:50 concentrations. The supernatant was discarded again and the precipitate was put in 2.5% glutaraldehyde stabilization buffer for 24 hr. The sample was then immobilized with sodium cacodylate buffer and osmium tetroxide. Sample dehydration was carried out in 50, 70, 90 and 100% ethanol (three times). The solution was stirred three times each for 20 min. The specimens were mixed in resin and propylene oxide mixtures at different ratios as follows: first, at a ratio of one to three resins and propylene oxide, then, at a ratio of one to one and finally, at a ratio of three to one (each for two times). The specimens were molded to polymerize at 60°C for two to three days and sectioned using an ultra-microtome. The sections were mounted on a copper grid impregnated with carbon form to protect the specimen from possible damages such as cracking and breaking. Uranyl acetate 1% and lead citrate were stained for optimum contrast and finally, the sample was dried. Finally, the sections were placed on the grid and examined by TEM.

**Tooth cleaning assay**

Four completely clean and sterile teeth were placed in BHI broth medium containing all isolated oral bacteria for one month, to form biofilm on the tooth. Then, to investigate the removal of biofilm formed on the teeth by plant mixture, the teeth were respectively exposed to the herbal mixture for 15, 35 and 45 sec (to determine the best time for biofilm removal), then the teeth were put in 0.1% crystal violet for 1 min, washed with distilled water and finally exposed for 95 sec to 95% ethanol to rinse the added color.

## Results

**Isolates**

The isolates were identified as *Streptococcus agalactia, Streptococcus pyogenes *and *Enterococcus faecalis* by microbiological methods.

**Acute toxicity results**

The essential oil of *S. officinalis, M. piperita* and *C. lippia* and extract of *E. purpurea *and* S. officinalis* did not produce any toxicity in rats.

**Anti-biofilm assay**

The results of the effect of *S. officinalis*, *L. citriodora, M. piperita* essential oils and coniferous and M. chamomilla extracts on oral bacteria showed that *S. officinalis*, *L. citriodora, M. piperita* essential oils were able to remove the biofilms created by these bacteria. However, coniferous and chamomile extracts were unable to counteract with the biofilm formed by these bacteria. Peppermint essential oil showed the highest ability to remove biofilm created by oral bacteria.

**MIC and MBC determination**

Due to the antimicrobial effects of the plants on the tested bacterial strains, considering their dilution, by combining them, a final product was obtained that had high inhibitory potency against oral microbes. This mixture was examined by light and electron microscopy. The obtained herbal mixture was tested on 7 strains of bacteria; the calculated MIC and MBC are presented in Table 3.

**Table 3:** MIC and MBC of plant mixture oral bacteria (mg/ml)

*Strains*	MBC	MIC
*Streptococcus agalactia*	0.00625	0.00625
*Streptococcus pyogenes*	0.0002	0.0002
*Streptococcus viridans*	0.006	0.0008
*Actinomyces viscousus*	0.012	0.0008
*Streptococcus sanguis*	0.006	0.0004
*Entrococcus faecalis*	0.012	0.0004
*Eikenella corrodens*	0.025	0.025

Against oral bacteria, the lowest MIC was 0.0002 mg/ml for *S. pyogenes* and the highest MIC was 0.25 mg/ml for *E. corrodens*. Also, the lowest calculated MBC (0.0002 mg/ml) was for *S. pyogenes* and the highest (0.025 mg/ml) belonged to *E. corrodens*.

**Light microscopy**

**Figure 1:**
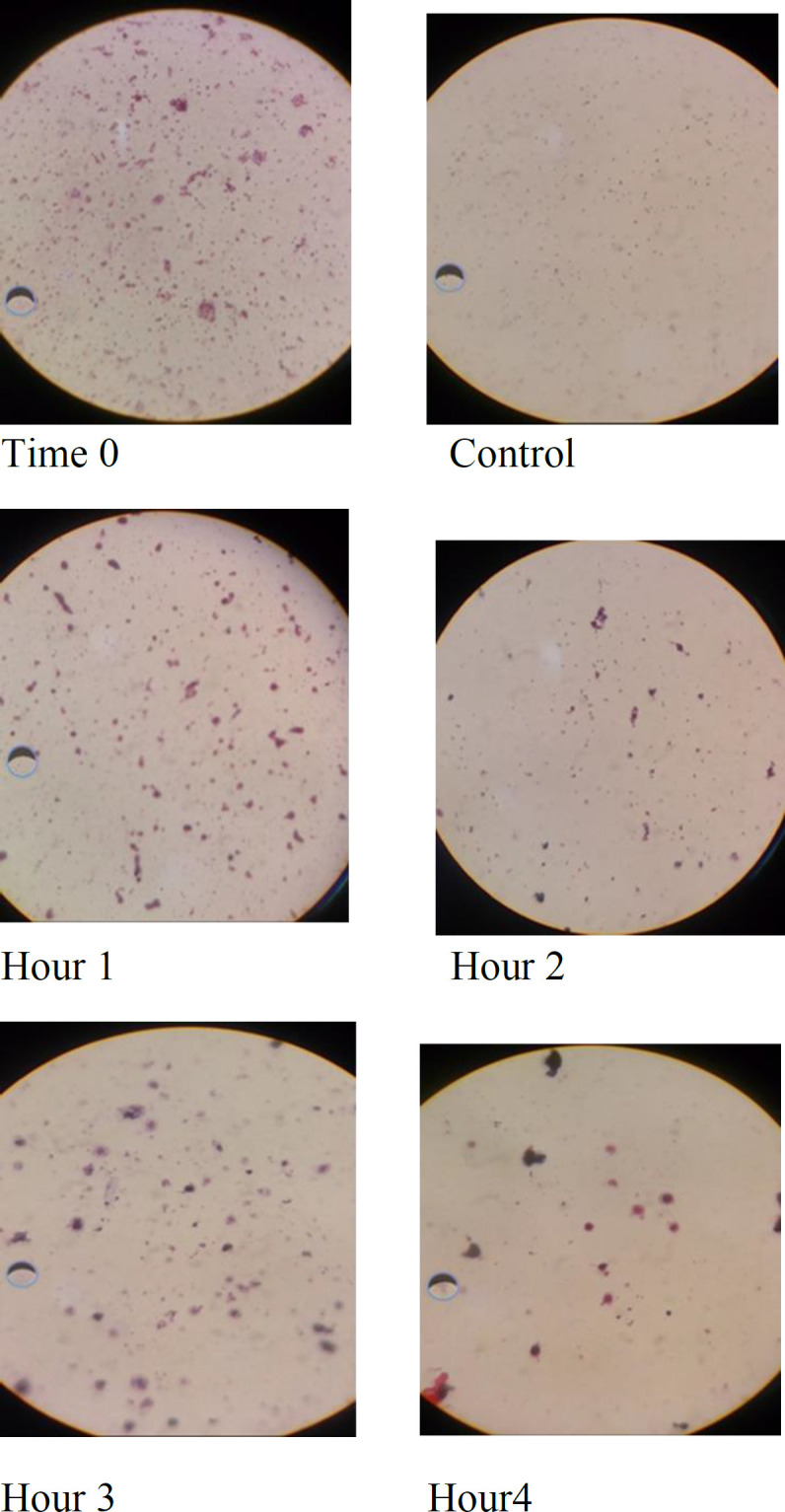
The cellular structure of *Streptococcus agalactiae*exposed to the plants mixture and the control bacteria

The mixture of the studied medicinal plants was exposed to each of the 7 oral microbes and examined by a light microscope following 0, 1, 2, 3 and 4 hr of exposure. The samples were Gram stained and examined by the light microscope (at 100X; Figure 1 and Table 4). The results of cell structure analysis of oral bacteria exposed to the plant extracts in comparison with the control at several exposure time-points, showed that cocci shape transformed into oval shape and cell destruction was observed. These effects increased with increasing exposure time

*Streptococcus agalactia, Estaphilococcus pyogenes, Actinomyces viscousus, Enterococcus faecalis and Eikenella corrodens* began structural degradation after one-hour contact but *Streptococcus viridans* degraded from beginning of experiment (time zero).

**Table 4:** Destruction effect of herbal material on bacterial strains in different exposure times

Strains	Times (hours)				
	0	1	2	3	4
*Streptococcus agalactia*	-	+	+	+	+
*Estaphilococcus pyogenes*	-	+	+	+	+
*Streptococcus viridans*	+	+	+	+	+
*Actinomyces viscousus*	-	+	+	+	+
*Streptococcus sanguis*	+	+	+	+	+
*Enterococcus faecalis*	-	+	+	+	+
*Eikenella corrodens*	-	+	+	+	+

**Electron microscopy**

Electron microscopy analysis of *Streptococcus sanguis* showed morphological disorganization after one-hour exposure. 

**Figure 2:**
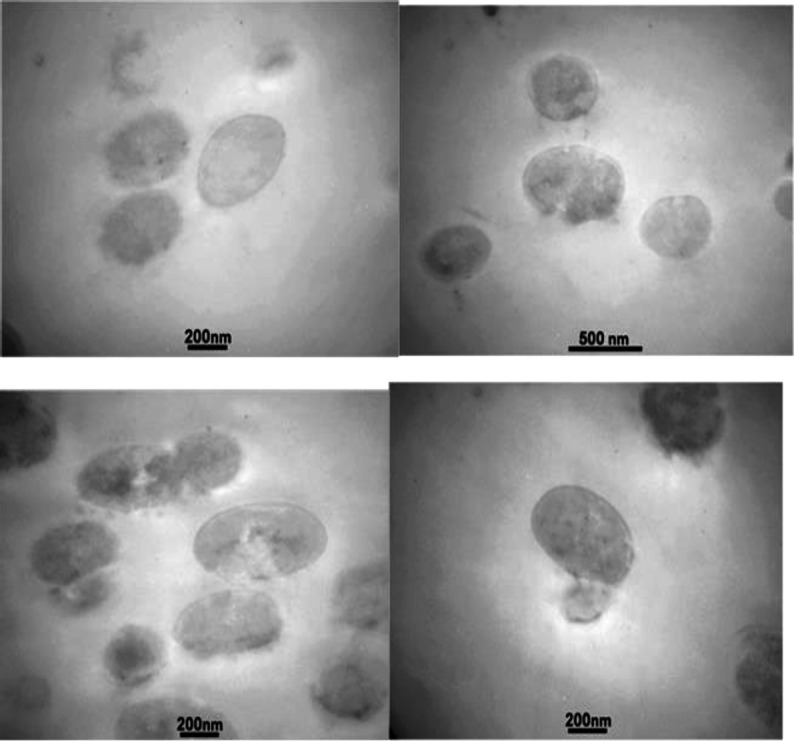
Bacterial cell abnormalities occurred after exposure to the herbal mixture by TEM

Invaginations, destruction, plasmolysis and severe cell wall alterations were observed in the cells following exposure to the herbal mixture. The cells changed from cocci to swollen, oval and other forms (Figure 2). 

The cell wall thickness was not changed in cells with intact peptidoglycan. In some cases, the peptidoglycan was fragmented and disrupted. The separation of the daughter cell from the mother was disproportionate. The cytoplasm of the cell was also released from the destroyed cell.

***In vivo*** **wound healing assay**

**Figure 3:**
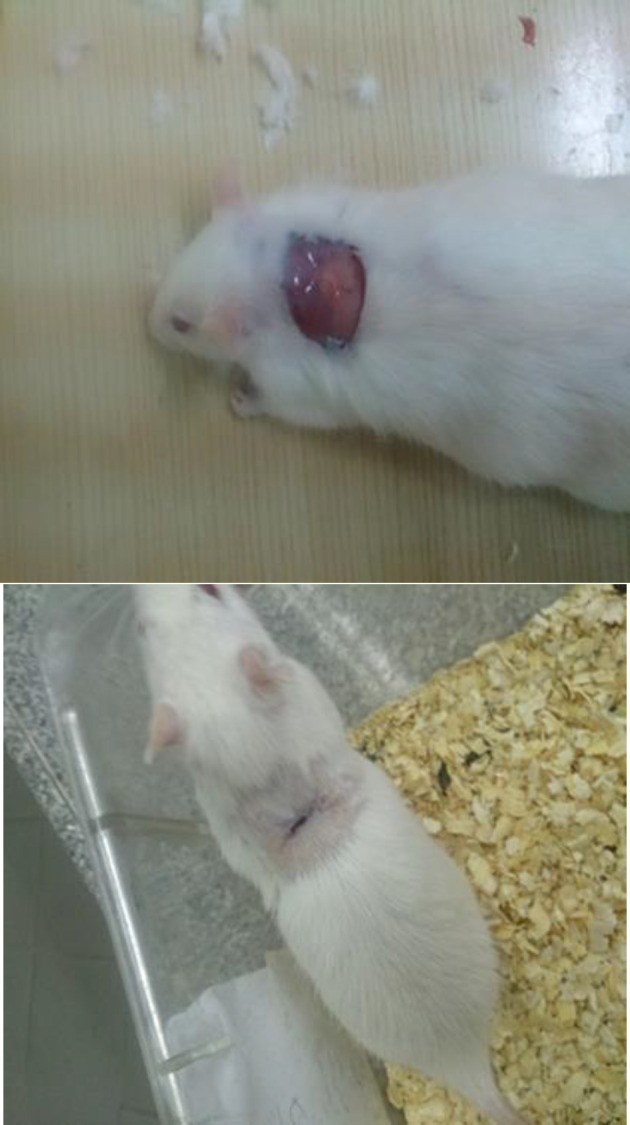
Wound healing assay on Wistar rats: Above: First day (immediately after scarring) Below: wound healing with herbal mixture

There was a significant increase in the rate of wound healing in rats treated with the herbal mixture to the control groups (p<0.05). In the treatment group, complete wound closure on day 4 and complete wound healing on day 21 were observed, whereas in the sham and control groups, complete recovery was observed after 30 days (Figure 3).

**Anti-biofilm assays on tooth**

**Figure 4:**
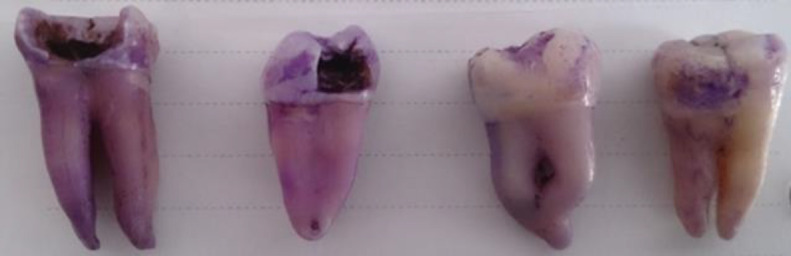
Staining of teeth after exposure to herbal material: (from left to right) Control (without treatment), and after 15, 30 and 45 seconds of exposure

Better results was obtained following the use of the herbal mixture in removal of biofilm from the tooth, compared to the the control (the control tooth was not exposed to the herbal mixture) and the exposure length of 45 sec was more suitable exposure time for biofilm removal (Figure 4).

**Assessment of genes expression**

**Figure 5:**
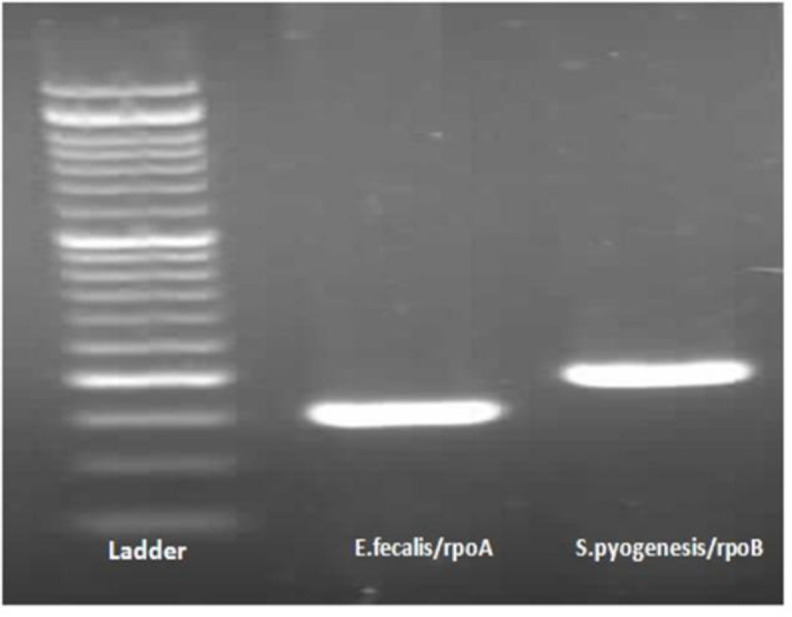
Electrophoresis on 1% agarose gel of universal PCR products. Left and right bonds belong to *E. faecalis* and *S. pyogenes*, respectively

The PCR reaction using primers designed based on genomic isolates of *E. faecalis* and *S. pyogenes* and their electrophoresis performed by 1% agarose gel is shown in Figure 4. *E. faecalis* was assayed by primers for *rpoB* gene that contained 151 bp fragments with *in vitro* amplification of the *rpoB* gene indicating the presence of this gene in the bacterium. To probe *S. pyogenes*, the PCR reaction was performed using a specific primer pair of the *rpoA* gene, which provided 198 fragments of fragment to amplify the *rpoA* gene, indicating the presence of this gene in *Streptococcus* bacterium (Figure 5). 

## Discussion

The studied herbal compounds were able to inhibit the growth of the examined strains, exert wound-healing properties in rats, without producing any undesirable effects, decompose the biofilm produced on tooth and deform the bacterial cell structure by causing extensive damage and a wide range of abnormalities. 

Tooth decay is one of the most important challenges for public health in different countries. Herbal compounds may have antibacterial properties and inhibitory effects on bacterial biofilm formation, and because of their low price, high availability, less side effects, they are candidate for being used as complementary agents along with common antibiotics and chemical materials (Giorgio et al., 1997 ▶) (Nurizadeh et al., 2004 ▶). 

According to the results of this study, the essential oils of *S. officinalis*, *M. piperita* and *L. citriodora* had biofilm inhibitory effects against the studied bacterial strains, but the extract of *E. purpurea* and *M. chamomilla* did not show biofilm inhibitory effects. 

Mohammed et al. (2019) ▶, assessed antibacterial effect of flower volatile oils from* Tecoma stans* (L.) and *Cassia javanica* (L.), as ornamental evergreen plants widely distributed in Egypt and traditionally used for oral hygiene purposes. They concluded that volatile oils of *C. javanica* had a potential antibacterial activity at low concentrations. This herbal material and *T. stans* had a strong activity against *Streptococcus mutans* and could be potential substituents to chlorhexidine.

Freire et al. (2019) ▶, studied anti-inflammatory effect of *Platymiscium floribundum* Vog. tree on periodontitis in rats in a pre-clinical trial. As a result, rats did not show signs of any toxicity and *P. floribundum* showed anti-inflammatory as well as anti-resorptive properties against periodontitis in rats.

In accordance with Freire et al. results, we concluded that our herbal mixture could heal skin wound in Wistar rats in 21 days without causing any undesirable effects.

Anti-adherence and antibacterial properties of Psidium, Mangifera and Mentha against *Streptococcus sanguinis* and *Streptococcus mutans*, were studied by Shafiei et al. (2016) ▶. Low MIC was achieved by the synergistic effect of the plant extracts. Different anti-adherence and antibacterial effect against *S. sanguinis* and *S. mutans* was shown to be due to different proportion of active materials of the herbal extracts.

The results of the present study also confirmed synergistic effects of herbal materials. It was concluded that the herbal mixture was able to decompose the biofilms produced by the studied bacteria. However, it must be noted that the herbal mixture used in the present work, consisted of diluted essential oils while Shafiei et al. used the herbal extracts. 

In the present study, the highest MIC value (0.025 mg/ml)of the herbal mixture was found against*E. corrodens* which was more effective in comparison with that achieved byIauk et al. (2003) ▶. The differences might be due to mode of preparation of herbal material (methanol extract against essential oil in our study) and bacterial genus used in two studies.

In Lang et al. study, the combined effects of essential oils of *M. piperita*, cinnamon and garlic on biofilm formation by *Pseudomonas aeruginosa* (PA01), were examined. Their results showed that adhesion and initiation of *P. aeruginosa* (PA01) biofilm production, was decreased. Oil was inhibited and at higher concentrations of these compounds completely removed the biofilm (Lang et al., 2016 ▶). Results of this study on the effect of peppermint oil on inhibition of *P. aeruginosa* biofilm formation are consistent with the results of the present study. 

Nicolai et al. (2009), worked on antimicrobial potential of ethanolic extract and essential oil of several plants from Lamiaceae family including Salvia, Mint, Thyme, Basil, etc. against resistant bacteria including *P. aeruginosa* and *E. coli*. They concluded that *P. aeruginosa* represented the highest MIC and MBC values against all extracts. All MIC and MBC values for P. aeruginosa were higher compared to E. coli.

In the current study, no toxicity was induced by the studied herbal materials which could be related to the small amounts of phenolic and flavonoid compounds in the extracts and essential oils of these plants (Galati and O'Brien, 2004 ▶).

In the present study, there was a significant difference in the percentage of wound healing and area reduction between the treatment and control groups. However, the complete recovery in the treatment group took 21 days, whereas in the control group, it took 30 days. These findings indicate the positive effects of the herbal mixture on deep cutaneous wound healing.

Based on *in vivo* and *in vitro* experiments It was concluded that the studied ethnomedicinal plants had antibacterial and antibiofilm properties on oral bacteria. 

It is recommended to continue the research on the activity of these herbs in prevention of tooth decay in clinical settings.

## Conflicts of interest

The authors have declared that there is no conflict of interest.
